# DCE-CT parameters as new functional imaging biomarkers at baseline and during immune checkpoint inhibitor therapy in patients with lung cancer – a feasibility study

**DOI:** 10.1186/s40644-024-00745-0

**Published:** 2024-08-13

**Authors:** Michael Brun Andersen, Aska Drljevic-Nielsen, Jeanette Haar Ehlers, Kennet Sønderstgaard Thorup, Anders Ohlhues Baandrup, Majbritt Palne, Finn Rasmussen

**Affiliations:** 1 Department of Radiology, Copenhagen University Hospital, Gentofte, Denmark; 2 Department of Radiology, Zealand University Hospital, Køge, Denmark; 3 Department of Radiology, Aarhus University Hospital, Skejby, Denmark; 4 Department of Radiology and Oncology, Aarhus University Hospital, Skejby, Denmark; 5 Department of Oncology, Zealand University Hospital, Køge, Denmark; 6 Radiology Department, Copenhagen University Hospital, Gentofte Hospitalsvej 1, 2900 Hellerup, Denmark; 7 Department of clinical medicine, Copenhagen University, Copenhagen, Denmark

**Keywords:** Tomography, Spiral computed, Carcinoma, Non-small-cell lung, Clinical oncology, Thorax, Immunotherapy

## Abstract

**Background:**

With the development of immune checkpoint inhibitors for the treatment of non-small cell lung cancer, the need for new functional imaging techniques and early response assessments has increased to account for new response patterns and the high cost of treatment. The present study was designed to assess the prognostic impact of dynamic contrast-enhanced computed tomography (DCE-CT) on survival outcomes in non-small cell lung cancer patients treated with immune checkpoint inhibitors.

**Methods:**

Thirty-three patients with inoperable non-small-cell lung cancer treated with immune checkpoint inhibitors were prospectively enrolled for DCE-CT as part of their follow-up. A single target lesion at baseline and subsequent follow-up examinations were enclosed in the DCE-CT. Blood volume deconvolution (BV_decon_), blood flow deconvolution (BF_decon_), blood flow maximum slope (BF_Max slope_) and permeability were assessed using overall survival (OS) and progression-free survival (PFS) as endpoints in Kaplan Meier and Cox regression analyses.

**Results:**

High baseline Blood Volume (BV_decon_) (> 12.97 ml × 100 g^−1^) was associated with a favorable OS (26.7 vs 7.9 months; *p* = 0.050) and PFS (14.6 vs 2.5 months; *p* = 0.050). At early follow-up on day seven a higher relative increase in BF_decon_ (> 24.50% for OS and > 12.04% for PFS) was associated with an unfavorable OS (8.7 months vs 23.1 months; *p* < 0.025) and PFS (2.5 vs 13.7 months; *p* < 0.018). The relative change in BF_decon_ (categorical) on day seven was a predictor of OS (HR 0.26, CI95: 0.06 to 0.93 *p* = 0.039) and PFS (HR 0.27, CI95: 0.09 to 0.85 *p* = 0.026).

**Conclusion:**

DCE-CT-identified parameters may serve as potential prognostic biomarkers at baseline and during early treatment in patients with NSCLC treated with immune checkpoint inhibitor therapy.

## Highlights

DCE-CT is a potential prognostic biomarker in NSCLC patients treated with immune checkpoint inhibitors.

High baseline BV_decon_ is associated with favorable OS and PFS

High relative increase in BF_decon_ day seven may be associated with unfavorable survival

## Background

Lung cancer remains the leading cause of cancer-related deaths worldwide [1]. In recent years new targeted therapies have emerged and the full effects of these treatments on global lung cancer survival remain unknown. Immune checkpoint inhibitors targeting programmed cell death receptor 1 (PD-1), was approved for clinical use in 2015 by the United States Food and Drug Administration [2, 3].

Currently, the response assessment to immune checkpoint inhibitor therapy relies on a combination of size criteria and temporal verification of changes. This was introduced with the immune response evaluation criteria in solid tumors (iRECIST) in October 2017 for conventional contrast enhanced computed tomography (CE-CT) [4]. Several research groups have investigated functional imaging for predictive and prognostic biomarkers. Most notably ^18^Flourodeoxyglucose positron emission tomography (^18^F-FDG PET) combined with CT to measure whole-body metabolic tumor volume (wbMTV) has shown promise [5]. Several retrospective studies have shown that wbMTV is associated with overall survival (OS) and progression free survival (PFS) in non-small cell lung cancer (NSCLC) [5–8]. A single prospective study investigating MTV and total lesion glycolysis (TLG) by Chardin et al. including seventy-five patients found that high MTV and TLG were associated with low OS and could predict early treatment discontinuation [9]. Furthermore, a single study by Park et al. retrospectively investigated early response assessment using ^18^F-FDG PET using the PET response criteria in solid tumors 1.0 (PERCIST) and found that peak standardized uptake value and MTV could predict progression [10]. Recent clinical experiments investigating novel targeted tracers for PD-1 using antibodies labelled with ^89^Zirconium have shown a positive correlation between tracer uptake and treatment response [11]. However, as the finding did not reach statistical significance further studies are needed prior to clinical implementation.

Compared to ^18^F-FDG PET/CT, conventional CE-CT are considerable cheaper and more accessible and by performing repeated scans over a single target lesion after injection of contrast media, changes in tumor contrast enhancement can be used to calculate perfusion parameters like arterial perfusion (deconvolution and max slope), permeability, blood volume, mean transit time and standardized perfusion [12, 13]. The method is referred to as Dynamic Contrast-Enhanced Computed Tomography (DCE-CT). In prior studies, several of these parameters have shown correlation with tissue vascularity and have been used for the quantification of tumor perfusion [14, 15]. Mains et al. showed that analyzing the histograms of perfusion parameter values within each voxel was the optimal approach to assess perfusion results; among seven different methods (median, mean, mode, standard deviation, interquartile range, skewness and kurtosis) the median value of the histogram had the best association to survival outcome [16]. A previous study by Lind et al. have shown that patients with NSCLC and partial response according to RECIST v1.1 have significantly higher baseline blood flow and blood volume than those with progressive disease. Furthermore, a decrease in blood flow after 3 and 6 weeks of therapy showed trends towards a longer progression free survival [17]. In the study, antiangiogenic treatment with sorafenib and erlotinib was used. Over the last few years treatment with immune checkpoint inhibitors have replaced conventional chemotherapy and antiangiogenic treatment as first- and second line treatment in select patients [18, 19]. To our knowledge no studies have explored the impact of DCE-CT on the evaluation of treatment response in patients with NSCLC treated with immune checkpoint inhibitor therapy.

The aim of the present feasibility study was to assess possible associations between baseline DCE-CT parameters and early changes during treatment with survival outcome in patients with NSCLC treated with immune checkpoint inhibitor therapy.

## Materials and methods

### Study population

This investigator-initiated, retrospective observational cohort study was approved by the Regional Ethical Committee (SJ-568) and the Danish Data Protection Agency (REG-02-2016). All the patients provided written informed consent. Inclusion criteria were patients with inoperable NSCLC, designated to receive first-, second-line or third line anti PD-1 or PD-L1 immune checkpoint inhibitor therapy. Well-defined primary tumor or metastases in the thoracic compartment suitable as a target for monitoring and analysis of DCE-CT parameters. Exclusion criteria were contraindications to iodinated contrast agents, events when DCE-CT was unobtainable, such as lack of cooperation during the examination, and patients that deviated from standard oncological treatment. A total of 33 patients were enrolled in this study.

Patient medical files were used to retrieve information about treatment and baseline clinical factors such as age, sex, tumor histology and PD-1 status.

### Study design

The potential study participants were identified at a multidisciplinary team conference. All patients initially received non-contrast-low dose CT of the chest for DCE-CT planning. This was followed by DCE-CT and CE-CT of the chosen lesion at baseline, day 7, day 30 and every three months for up to 12 months, whereupon the patients continued with standard follow-up. In case of tumor progression or discontinuation of immune checkpoint inhibitor therapy of other causes, patients reverted to standard follow-up with CE-CT every three months.

### Selection of target lesion for DCE-CT

An experienced thoracic radiologist with 12 years of experience was present for the selection of the single target lesion most optimal for functional CT at all baseline scans. Potentially, both primary tumors and metastases can be chosen as target lesions. However, in the present study, only primary tumors were selected. Only solid lesions could be chosen, as it was expected that groundglass components would affect the DCE-CT parameters. Lesions in the middle and upper lung zones away from the diaphragm were favored to minimize the risk of motion artefacts. No upper size limit for tumors was used, and as such in three cases only part of the tumor was enclosed in the DCE-CT volume of 8 cm. In these cases, areas with high density on the low-dose scan were favored to avoid potential necrotic areas.

### CE-CT and DCE-CT

DCE-CT was initially performed over a single target lesion followed by CE-CT of the thorax and upper abdomen. The current study was limited to the DCE-CT data.

All scans were performed using the iCT 256 CT system (Philips, Best, The Netherlands). DCE-CT was performed after an injection of 60 ml of Iomeprol (Bracco Imaging, Milano, Italy) 320 mg I/ml at 6 ml/sec. Scans were started 1 s after the initiation of contrast injection. The scan parameters were 100 kVp, 100 mAs, pitch 0.9, rotation speed 0.27 ms, collimation 0.625 × 256 with a total Z-axis coverage of 8 cm. For the first 25 s scans were performed with a 2 s interval. To decrease radiation dose after the first 13 cycles the interval was increased to 4 s and scans continued until 57 s after contrast injection for a total of 21 scan cycles. All images were reconstructed with a field of view of 350 mm, a matrix of 512 × 512, a slice thickness of 1 mm, and soft reconstruction kernel providing a voxel size of 0.68 × 0.68 × 1 mm.

### Delineation and DCE-CT assessments

DCE-CT analysis was performed using the prototype software program Advanced Perfusion and Permeability Application (APPA) (Philips, Best, The Netherlands). The software allows an analysis of the entire volume of the target lesion and combined with repeated scan cycles it provides an assessment in 4-dimensions. The DCE-CT scan data were initially loaded into the software; subsequently, non-rigid registration was used for spatial filtration and motion correction. To obtain the highest perfusion parameters, target lesions were assessed at arterial peak enhancement (PE). The morphologic series and relevant perfusion series were generated and displayed at PE. These data were loaded into Intellispace 6.0 Multimodality Tumor Tracking, (Philips, Best, The Netherlands). Using a semi-automatic 3D tool, the target lesion was delineated at the morphologic series using axial, coronal and sagittal multiplanar reformats defining the volume of interest (VOI). All analyses were performed by a thoracic radiologist with 12 years of experience, who was blinded to clinical information and outcome.

The APPA provides several perfusion parameters including: blood volume using the deconvolution model (BV_decon_, ml × 100 g^−1^), blood flow using deconvolution (BF_decon_, ml × min^−1^ × 100 g^−1^) and maximum slope model (BF_max slope_, ml × min^−1^ × 100 g^−1^), time to peak (TTP, sec), mean transit time (MTT, sec), and permeability surface area product by the Patlak model (ml × min^−1^ × 100 g^−1^) [12, 13].

Dynamic data combined with the VOI were loaded and analyzed in MATLAB (v. R2015b, MathWorks Inc., Natick, MA, USA), where histogram values of the perfusion parameters were extracted using in-house software can be shared upon reasonable request. The median values were calculated for each histogram, as this has previously been shown to be the most reproducible and used for statistical analysis [16].

### Statistical analysis

Progression-free-survival (PFS) was defined as the time between study inclusion and progression according to the iRECIST, clinical assessment, or cancer-related death, whichever came first. Overall survival (OS) was defined as the time between inclusion and death. The relative change in the percentage of perfusion parameters at day seven and day thirty was calculated using the following equation:

$$\frac{Value\, at \,treatment \,time \,point-Value \,at \,Baseline)}{Value \,at \,Baseline} X 100$$

All perfusion parameters and relative changes in percentage were graphically checked for Gaussian distribution by Q-Q plots. Normally distributed parameters are presented as mean (standard deviation). In the case of an absent normal distribution, data are presented as median (range).

For the mean and median values of all perfusion parameters, as well as for all relative changes in percent for those parameters, optimal thresholds were determined by the cutp function in R to split the perfusion parameters into categorical values [20].

The univariate Cox proportional hazards model was used to assess the association of baseline DCE-CT parameters as well as the relative change in percentage at day seven and day thirty as both categorical and continuous variables with PFS and OS as endpoints. Results were expressed as a hazard ratio (HR) with 95% confidence intervals (CI95). The assumptions of proportional hazards were tested graphically using scaled Schoenfeld residuals against transformed time.

For the parameters that were predictors of survival, Kaplan-Meier curves were generated based on the optimal threshold, and the difference between survival curves for the group above or below the threshold was evaluated using a nonparametric log-rank test. The median follow-up was calculated using the reverse Kaplan-Meier method.

All analyses were conducted using the R software package 4.0.3 (R Core Team, 2020). In R the following packages were used: Dplyr (version 1.0.3, 2021), Survival (version 3.2-7, 2020), Survminer (version 0.4.9, 2021), SurvMisc (version 0.5.5, 2018), Prodlim (version 2019.11.13) and Publish (version 2020.12.23) [21–27]. All statistical tests were two-sided, and *p* values below 0.05 were considered statistically significant.

## Results

### Patients

**Fig. 1:**
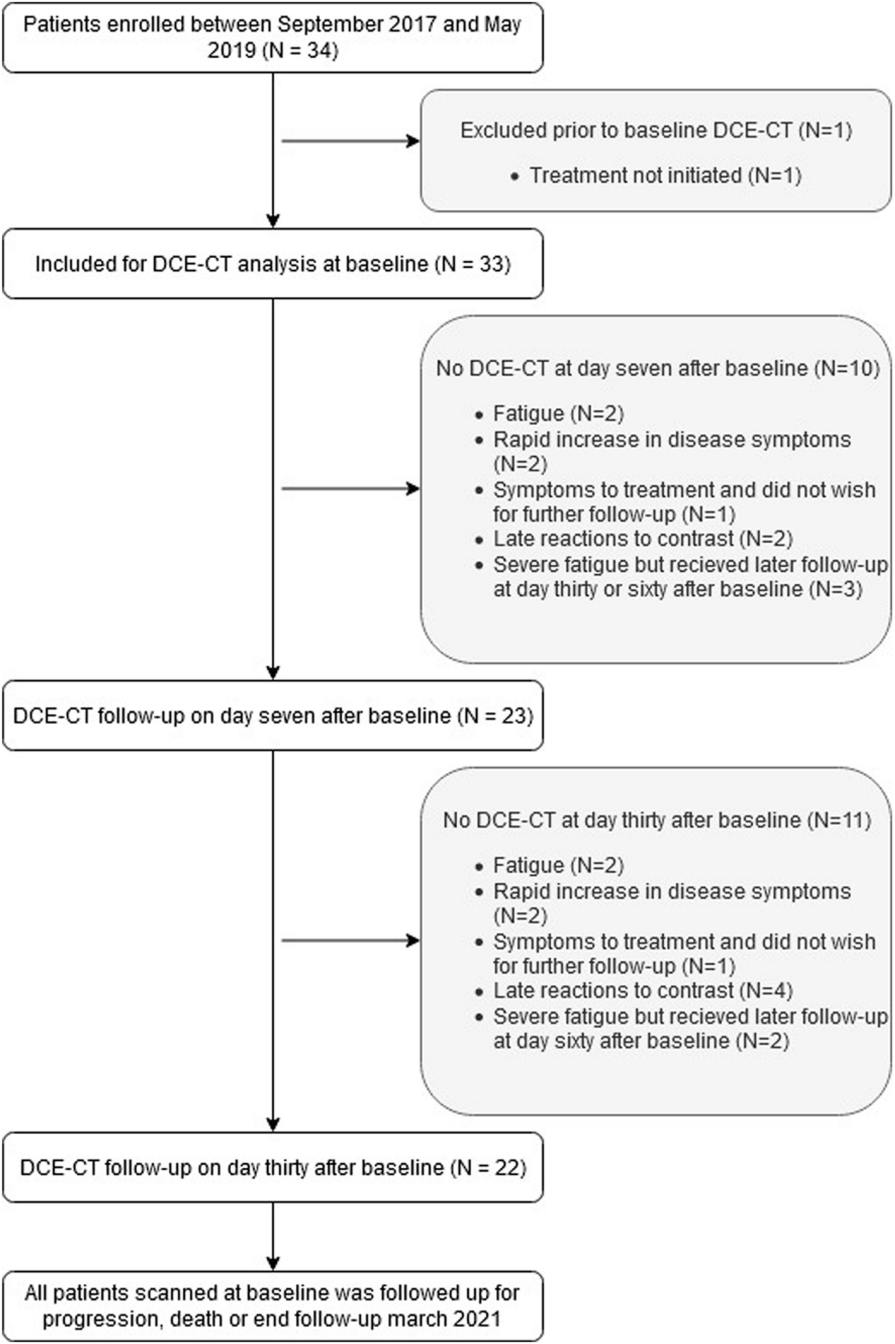
Flowchart of patient inclusion and study design

A total of 34 patients, all diagnosed with primary lung cancer and referred for treatment with anti PD-1 immune checkpoint inhibitor therapy, were prospectively included between September 2017 and May 2019. One patient was excluded because oncological treatment was not initiated as the patient received argon beam treatment for a tumor in the trachea. At baseline, 33 patients (15 males and 18 females; mean age 69.9 ± 7.6) were included in the final analysis. Table 1 lists the basic demographics, radiation dose, clinical stage, immune checkpoint inhibitor therapy agent, treatment line, and pathology. At day seven 23 patients underwent follow-up with DCE-CT, and on day thirty, 22 patients were followed up (Fig. 1). The median follow-up time in alive patients was 29.23 months (CI:26.63 to 35.30 months).

**Table 1:** Patient demographics: age, overall survival, progression-free survival, sex, radiation dose for dynamic contrast enhanced CT, pathology, clinical stage, immunotherapy agent used and line of treatment

**Characteristics**	**Description**	**Total (*N* = 33)**
**Age**	Years of age	69.9 (CI95% 67.2 to 72.6)
**OS**	Months	21.7 (Min: 1 to Max: 42)
**PFS**	Months	6.1 (Min: 1 to Max: 42)
**Sex**	Male	15
	Female	18
**Radiation dose**	DLP	970.6 (CI95% 913.8 to 1027.4)
**Primary tumor pathology**	Adenocarcinom	25
	Squamous Cell Carcinoma	6
	Unclassified NSCLC	2
**Clinical stage**	IIIB	1
	IIIC	1
	IVA	18
	IVB	8
	Recidivation	5
**Immunotherapy agent**	Pembrolizumab	31
	Nivolumab	1
	Atezolizumab	1
**Line of treatment**	First	25
	Second	5
	Third	3

*DLP* Dose Length Product, *NSCLC* Non-Small Cell Lung Cancer

Of the initial 33 patients, only 5 had undergone DCE-CT during the entire 12-months follow-up, whereas 11 patients died, 12 patients developed progression. Five patients experienced adverse reactions to treatment with immune checkpoint inhibitors or the combination of immune checkpoint inhibitor therapy and contrast agents. As a result, they did not receive any further DCE-CT scans but reverted to ordinary follow-up.

### Assessment of distribution

All DCE-CT parameters showed non-Gaussian distributions. This included subgroup analysis based on the optimal cutoff-points for the individual parameters and relative changes in percent.

### Intra/inter tumor heterogeneity

**Fig. 2:**
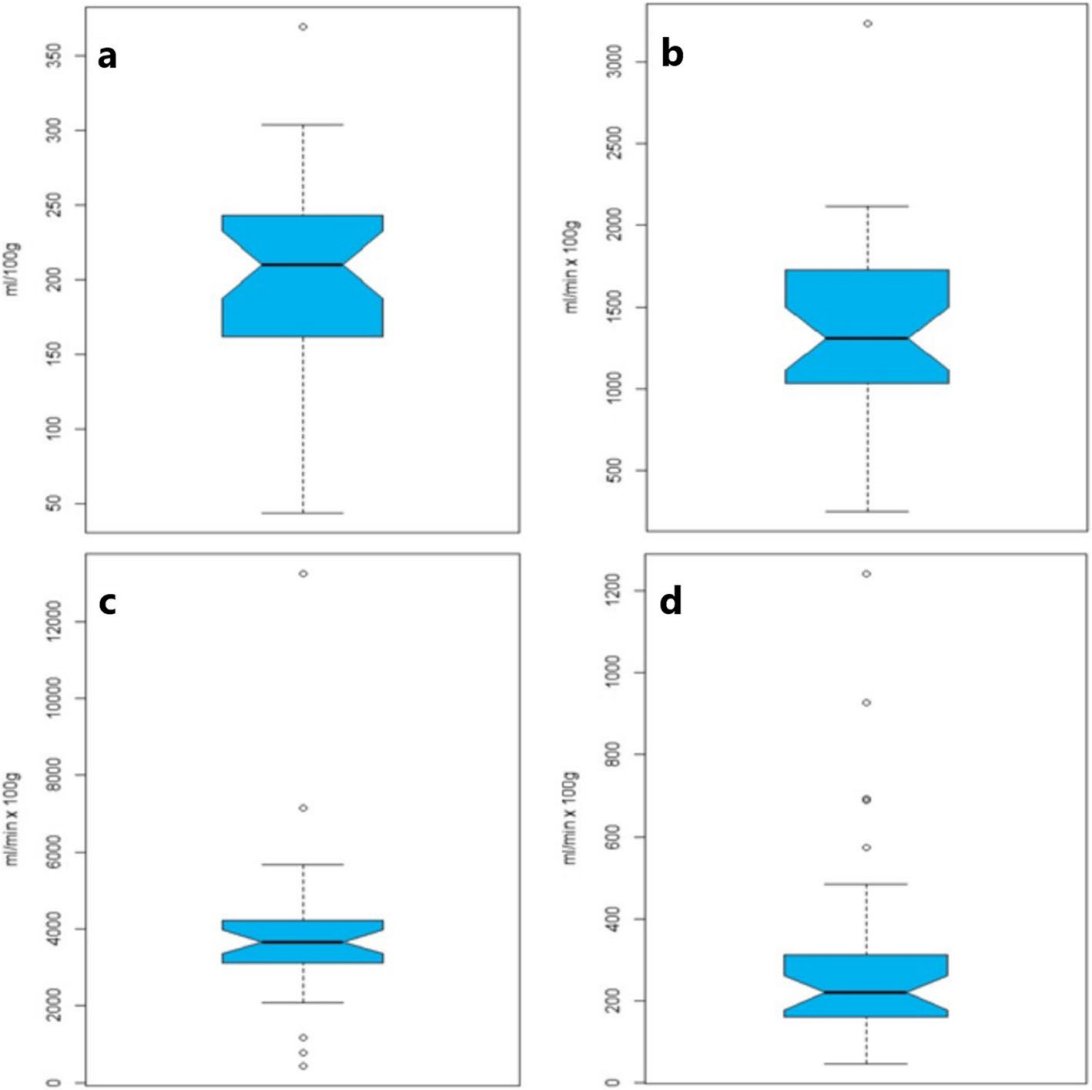
Box plots of the range calculated as the minimal value subtracted from the maximum value of DCE-CT parameters for the individual lesions. Blood volume (**a**), blood flow deconvolution (**b**), blood flow maximum slope (**c**) and permeability (**d**)

The median histogram width (The difference between the minimum and maximum values of the histograms) at baseline for Blood Volume (BV_decon_) was 209.97 ml × 100 g^−1^ (43.95 to 369.34 ml × 100 g^−1^), for Blood Flow deconvolution (BF_decon_) 1307.54 ml × min^−1^ × 100 g^−1^ (250.02 to 3234.66 ml × min^−1^ × 100 g^−1^), for BF Max-slope (BF_Max-slope_) 3667.83 ml × min^−1^ × 100 g^−1^ (443.72 to 13,242.39 ml × min^−1^ × 100 g^−1^) and for Permeability 220.35 ml × min^−1^ × 100 g^−1^ (47.01 to 1240.77 ml × min^−1^ × 100 g^−1^) (Fig. 2).

### Optimal cut-points and DCE-CT parameters

**Fig. 3:**
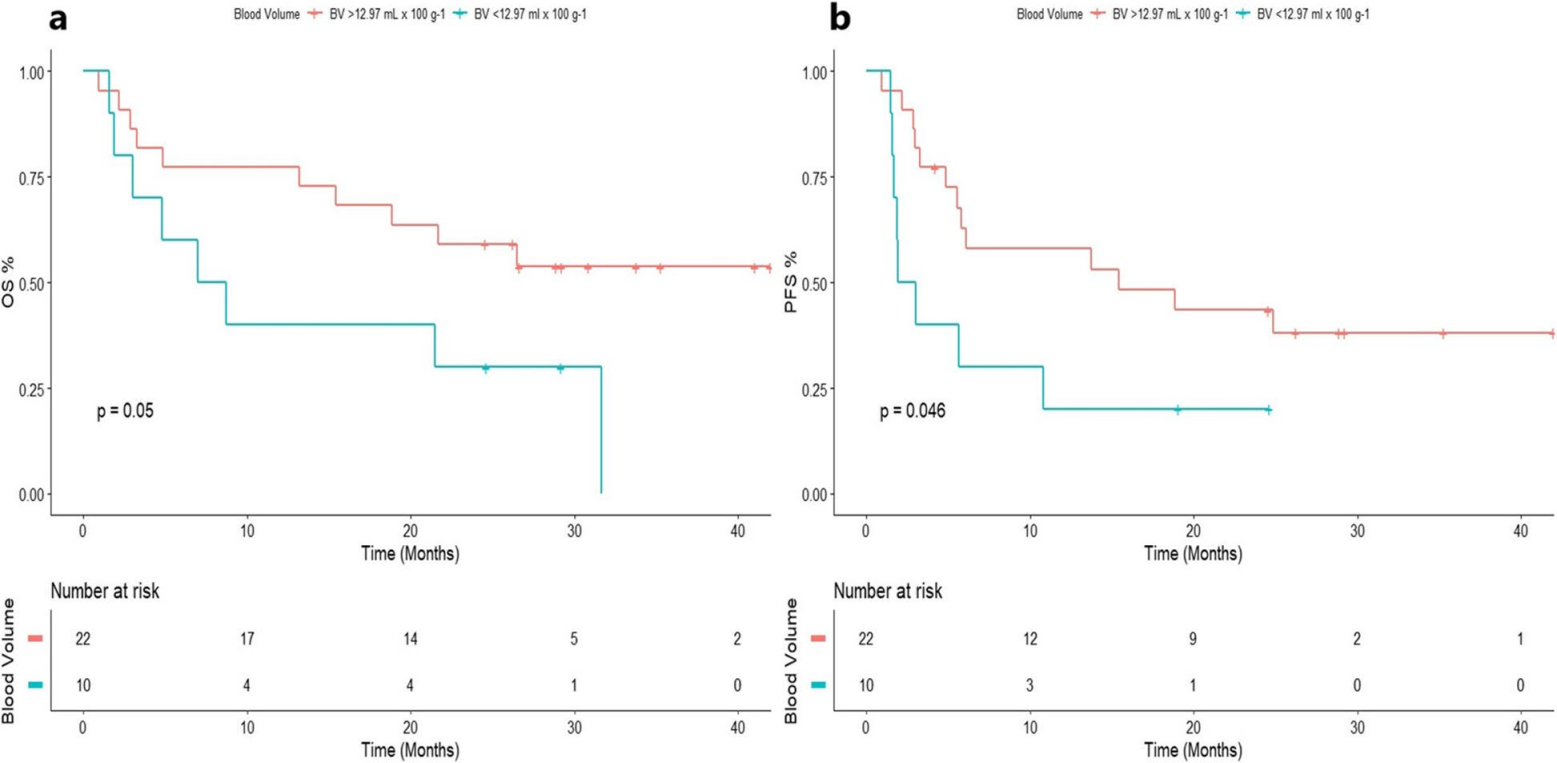
Baseline high blood volume is associated with favorable patient outcome. **a** Overall survival and **b** progression-free survival

The DCE-CT parameters dichotomized by the optimal cutoff-points are presented in Table 2. We found that high baseline Blood Volume (BV_decon_) (> 12.97 ml × 100 g^−1^) was associated with a favorable OS (26.7 vs 7.9 months; *p* = 0.050) and PFS (14.6 vs 2.5 months; *p* = 0.046) (Fig. 3). No other DCE-CT parameters showed statistically significant associations. However, high baseline blood flow deconvolution (BF_decon_) (> 75.02 ml × min^−1^ × 100 g^−1^) showed a trend towards association with a favorable PFS (9.92 months vs 2.45 months; *p* = 0.090).

**Table 2:** Dynamic contrast enhanced CT parameters associated with patient outcome by log rank tests on baseline, day seven and day thirty of follow-up

**Perfusion Parameter**	**Optimal cut-off OS**	**Range**	**Median Os, mo**	**Log-Rank (P)**	**Optimal cut-off PFS**	**Range**	**Median PFS, mo**	**Log-Rank (P)**
**Baseline (*n* = 32)**								
Blood Volume	≤ 12.97 (*n* = 10)	5.34 to 11.77	7.87	0.050	≤ 12.97 (*n* = 10)	5.34 to 11.77	2.48	0.046
	> 12.97 (*n* = 22)	12.97 to 120.46	26.37		> 12.97 (*n* = 22)	12.97 to 120.46	14.58	
Blood Flow (Deconvolution)	≤ 75.02 (*n* = 6)	43.93 to 75.02	25.4	0.11	≤ 75.02 (*n* = 6)	43.93 to 75.02	2.45	0.09
	> 75.02 (*n* = 26)	79.23 to 1314.05	6.78		> 75.02 (*n* = 26)	79.23 to 1314.05	9.92	
Blood Flow (Max slope)	≤ 302.94 (*n* = 24)	112.04 to 302.94	25.4	0.21	≤ 302.94 (*n* = 24)	112.04 to 302.94	5.57	0.29
	> 302.94 (*n* = 8)	579.03 to 2465.04	21.5		> 302.94 (*n* = 8)	579.03 to 2465.04	20	
Permeability	≤ 28.78 (*n* = 17)	6.19 to 28.78	14.32	0.11	≤ 31.29 (*n* = 20)	6.19 to 31.29	3.75	0.27
	> 28.78 (*n* = 15)	29.44 to 63.29	26.23		> 31.29 (*n* = 12)	34.75 to 63.29	15.43	
**Relative change from baseline to day 7 (*n* = 23)**								
Blood Volume	≤ 7.31 (*n* = 15)	-64.39 to 7.31%	24.6	0.2	≤ 5.61 (*n* = 14)	-64.39 to 5.61%	5.73	0.35
	> 7.31 (*n* = 8)	8.81 to 61.49%	10.97		> 5.61 (*n* = 9)	7.31 to 61.49%	2.87	
Blood Flow (Deconvolution)	≤ 24.50 (*n* = 20)	-72.81 to 24.50%	23.12	0.025	≤ 12.04 (*n* = 17)	-72.80 to 12.04%	13.73	0.018
	> 24.50 (*n* = 3)	39.24 to 69.05%	8.73		> 12.04 (*n* = 6)	15.92 to 69.05%	2.47	
Blood Flow (Max slope)	≤ -43.58 (*n* = 19)	-76.01 to -43.58%	27.72	0.16	≤ -43.58 (*n* = 19)	-76.01 to -43.58%	20	0.16
	> -43.58 (*n* = 4)	-35.72 to 143.49%	18.83		> -43.58 (*n* = 4)	-35.72 to 143.49%	4.87	
Permeability	≤ -0.25 (*n* = 7)	-41.69 to -0.25%	15.43	0.31	≤ 48.82 (*n* = 19)	-41.69 to 48.82%	5.57	0.22
	> -0.25 (*n* = 13)	0.52 to 107.17%	21.67		> 48.82 (*n* = 3)	58.81 to 107.17%	29.23	
**Relative change from baseline to day 30 (*n* = 22)**								
Blood Volume	≤ 7.67 (*n* = 10)	-90.09 to 7.67%	18.5	0.69	≤ -7.60 (*n* = 13)	-90.09 to -7.60%	4.2	0.33
	> 7.67 (*n* = 12)	10.35 to 103.96%	21.7		> -7.60 (*n* = 9)	7.67 to 103.96%	6.1	
Blood Flow (Deconvolution)	≤ 12.96 (*n* = 9)	-79.27 to 12.96%	15.43	0.14	≤ 4.07 (*n* = 9)	-79.27 to 4.07%	4.2	0.12
	> 12.96 (*n* = 12)	13.40 to 47.11%	25.53		> 4.07 (*n* = 12)	12.96 to 47.11%	8.47	
Blood Flow (Max slope)	≤ 8.98 (*n* = 14)	-94.79 to 8.98%	14.32	0.064	≤ 24.59 (*n* = 17)	-94.79 to 24.59%	5.57	0.55
	> 8.98 (*n* = 7)	18.10 to 49.11%	29.17		> 24.59 (*n* = 4)	25.10 to 49.11%	14.83	
Permeability	≤ -1.11 (*n* = 7)	-65.41 to -1.11%	29.17	0.13	≤ 8.66 (*n* = 12)	-65.41 to 8.66%	5.95	0.23
	> -1.11 (*n* = 13)	-0.40 to 1166.83%	18.83		> 8.66 (*n* = 8)	19.40 to 1166.83%	3.15	

*OS* Overall Survival, *PFS* Progression Free Survival

**Fig. 4:**
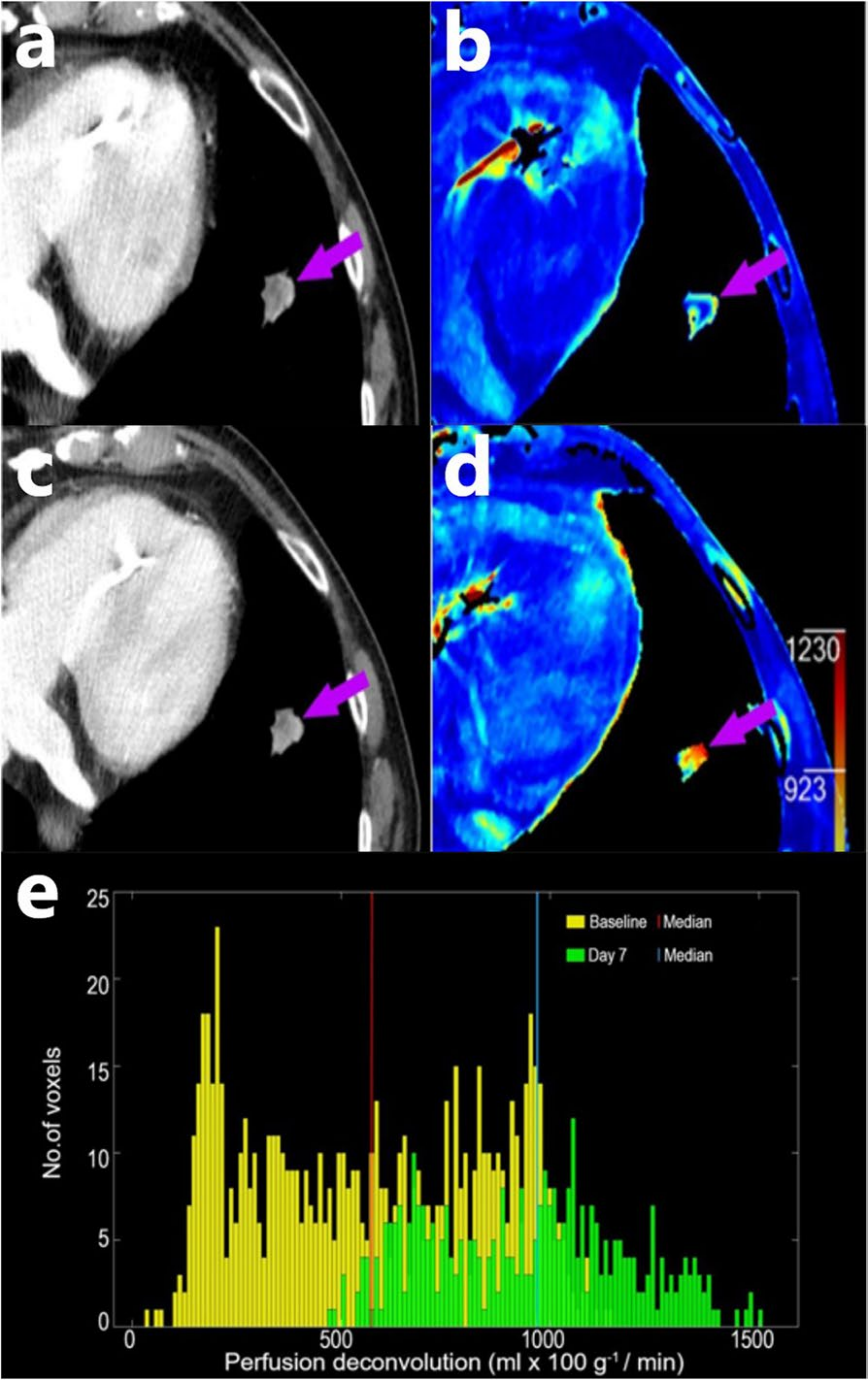
A patient with NSCLC in the left lung on contrast-enhanced CT at baseline (**a**) and seven days after baseline (**b**); and the corresponding DCE-CT BF_decon_ map at baseline (**c**) and seven days after baseline (**d**). The BF_decon_ histogram of the tumor depicts an increase of 69% in median BF_decon_ values from baseline (573 ml x min^−1^ × 100 g^−1^) to day seven (968 ml x min^−1^ × 100 g^−1^). BF_decon_ = Blood flow deconvolution, DCE-CT = Dynamic contrast enhanced computed tomography, NSCLC = Non-small cell lung cancer

At early follow-up on day seven a higher relative increase in BF_decon_ (> 24.50% for OS and > 12.04% for PFS) was associated with an unfavorable OS (8.7 months vs 23.1 months; *p* < 0.025) and PFS (2.5 vs 13.7 months; *p* < 0.018) (Fig. 4).

Relative changes in DCE-CT parameters at day 30 only showed a trend towards high relative increase in BF Max-slope (BF_Max-slope_) (> 8.98%) with a favorable OS (29.2 vs 14.3 months; *p* = 0.064).

In addition, MTT and TTP were analyzed but showed no association with either OS or PFS at any time point.

### Cox regression analysis of DCE-CT parameters at baseline and follow-up

The hazard ratios of both continuous and categorical DCE-CT parameters are presented in Table 3 for both OS and PFS. At baseline, low BV_decon_ showed a trend toward unfavorable PFS (HR 2.44, CI95:0.99 to 6.02; *p* = 0.053) and OS (HR 2.48, CI95:0.99 to 6.34; *p* = 0.057).

**Table 3:** Univariate cox regression for the effect of dynamic contrast enhanced CT (DCE-CT) parameters at baseline and day seven of follow-up on patient outcome

**Perfusion Parameter**	**Hazard Ratio (95% CI) OS**	***P***	**Hazard Ratio (95% CI) PFS**	***P***
**Baseline**				
Blood Volume	0.99 (0.97 to 1.01)	0.300	0.99 (0.98 to 1.01)	0.592
Blood Volume Categorical	2.48 (0.97 to 6.34)	0.057	2.44 (0.99 to 6.02)	0.053
Blood Flow (Deconvolution)	0.99 (0.99 to 1.00)	0.210	0.99 (0.99 to 1.00)	0.485
Blood Flow (Deconvolution) Categorical	2.28 (0.97 to 6.34)	0.121	2.38 (0.86 to 6.62)	0.095
Blood Flow (Max-slope)	0.99 (0.99 to 1.00)	0.229	0.99 (0.99 to 1.00)	0.382
Blood Flow (Max-slope) Categorical	2.15 (0.62 to 7.39)	0.226	1.71 (0.63 to 4.66)	0.294
Permeability	1.00 (0.97 to 1.04)	0.876	1.00 (0.97 to 1.04)	0.628
Permeability Categorical	0.47 (0.18 to 1.21)	0.117	0.79 (0.33 to 1.89)	0.602
**Relative change from baseline to day 7**				
Blood Volume	1.00 (0.98 to 1.01)	0.940	1.00 (0.98 to 1.01)	0.708
Blood Volume Categorical	0.50 (0.17 to 1.48)	0.212	0.62 (0.23 to 1.68)	0.350
Blood Flow (Deconvolution)	1.00 (0.99 to 1.02)	0.499	1.01 (0.99 to 1.02)	0.484
Blood Flow (Deconvolution) Categorical	0.26 (0.06 to 0.93)	0.039	0.27 (0.09 to 0.85)	0.026
Blood Flow (Max-slope)	1.00 (0.99 to 1.02)	0.367	1.00 (0.99 to 1.01)	0.646
Blood Flow (Max-slope) Categorical	0.26 (0.03 to 1.96)	0.190	0.36 (0.03 to 1.96)	0.181
Permeability	0.99 (0.98 to 1.01)	0.481	1.00 (0.98 to 1.01)	0.640
Permeability Categorical	1.71 (0.59 to 4.95)	0.320	3.35 (0.44 to 25.64)	0.245

For relative changes from baseline to day seven only BF_decon_ as a categorical variable was a predictor of OS (HR 0.26, CI95:0.06 to 0.93; *p* = 0.039) and PFS (HR 0.27, CI95:0.09 to 0.85; *p* = 0.026). The remaining variables were not associated with OS or PFS (Table 3).

## Discussion

In this feasibility study, we found that high baseline BV_decon_ might be associated with favorable OS and PFS. Furthermore, we found that a relatively higher decrease in BF_decon_ during the early follow-up (seven days after baseline) might be associated with a more favorable survival outcome.

To our knowledge, no study has reported the impact of DCE-CT in patients with lung cancer treated with immune checkpoint inhibitor. However, several studies have investigated DCE-CT in patients with NSCLC treated with conventional chemotherapy or chemoradiotherapy [28–31]. Sudarski et al. showed no difference in BV, BF_decon_, or permeability in patients treated with conventional chemotherapy using response according to iRECIST as the endpoint. Similarly, DCE-CT parameters showed no correlation with OS [28]. Venkat et al. found that patients with NSCLC responding to conventional chemotherapy had higher baseline BF and permeability than non-responders (*p* = 0.047 and 0.028, respectively). Post-treatment, a greater decrease in BF values was noted among responders than among non-responders, although the results were not statistically significant [29]. Wang et al. showed similar results, in which patients responding to chemoradiation therapy had significantly higher baseline BF than non-responders. An increase in permeability-surface area was a significant predictor of both longer OS (10.6 vs 19.3 months, *p* = 0.004) and PFS (4.7 vs 19.0 months, *p* < 0.001) [30]. In a recent study López et al. showed that a significant decrease in BV (21%, *p* = 0.006) and MTT (17%, *p* = 0.031) was found in patients with partial response to treatment with platinum derivates traditionally used as chemotherapy for NSCLC [31]. Prior studies have employed measurements of selected region of interests within the target lesion. In this study, the entire volume of the lesion was segmented, thereby accounting for the intratumoral heterogeneity.

In line with our findings, high baseline BV showed similar results in other tumor types and treatments. Recently, Drljevic-Nielsen et al. identified high baseline BV as a true independent prognostic factor for longer OS (HR 0.49, CI 95% 0.30 to 0.78; *p* = 0.003) and PFS (HR 0.64, CI95% 0.42 to 0.97; *p* = 0.036) in patients with metastatic renal cell carcinoma treated with antiangiogenetic drugs or immune checkpoint inhibitors [32]. Similar to the findings of the present study, Drljevic-Nielsen et al. found that high baseline BV was associated with long OS (42.2 vs 22.4 months, *p* = 0.001) and PFS (12.5 vs 5.6 months, *p* = 0.003). Similar to our results, Mains et al. showed that a higher relative reduction in BV and BF at weeks 5 and 10 was associated with a more favorable survival outcome [33].

BV has previously been correlated with tumor micro vessel density and is a reflection of vascularity [13]. The median BV_decon_ at baseline in the current study was 15.14 mL × 100 g^−1^ with a wide range (5.34 to 120.46 mL × 100 g^−1^). Furthermore, the median histogram width for BV_decon_ of 209.97 ml × 100 g^−1^ (43.95 to 369.34 ml × 100 g^−1^), for BF_decon_ of 1307.54 ml × min^−1^ × 100 g^−1^ (250.02 to 3234.66 ml × min^−1^ × 100 g^−1^), for BF_Max-slope_ of 3667.87 ml × min^−1^ × 100 g^−1^ (443.72 to 13,242.39 ml × min^−1^ × 100 g^−1^) and for permeability of 220.35 ml × min^−1^ × 100 g^−1^ (47.01 to 1240.77 ml × min^−1^ × 100 g^−1^) (Fig. 2). These reflect a high degree of intratumoral heterogeneity, which is further illustrated in broad histograms (Fig. 3). The wide range of the median histogram width for the various parameters reflects a high degree of intertumoral heterogeneity. In the present study, high BV_decon_, as a measure of high vascularity, was correlated with a favorable outcome. This is consistent with prior studies where tumor cells have been shown to survive in microenvironments with hypoxia (low oxygenation), which subsequently leads to resistance to radiotherapy for the tumors [34]. It appears that a similar mechanism may be related to both chemotherapy and immune checkpoint inhibitor therapy.

Currently, PD-L1 expression in lung tumors is used to allocate patients to either immune checkpoint inhibitor therapy or conventional chemotherapy as the first-line treatment. However, PD-L1 status alone is not sufficient for treatment with anti-PD-1 based therapies, as a response can be seen in patients without PD-L1 expression and no response can be seen in patients with high-grade PD-L1 [35]. Therefore, new biomarkers are needed to determine prognosis prior to treatment and to evaluate response during ongoing treatment, DCE-CT could have that potential.

DCE-CT follow-up for oncological treatment in clinical practice is rarely performed for several reasons. Coverage of only a small section of the anatomy and often only enclosing one or a few of the target lesions the technique lacks the ability to account for inter tumoral heterogeneity in lung cancer patients [36]. The selection of the lesion for analysis is dependent on the radiologist performing the examination and can be due to the heterogeneous nature of the lesions affecting the measurements for individual patients. In addition, five patients in the current study experienced adverse effects to the combination of iodinated contrast agents and immune checkpoint inhibitor therapy and were admitted to the oncology ward within twenty-four hours of receiving the DCE-CT with a clinical image of sepsis, but all recovered with supportive treatment within the following twelve to twenty-four hours. Furthermore, patient collaboration and radiation dose to patients are major concerns. For the latter, we employ a technique with a decreased amount of scan cycles after the initial arterial peak decreasing the overall radiation dose so it corresponds to a standard CT scan of the thorax and upper abdomen (970.6 DLP, CI 95% 913.8 to 1027.4). The short intervals between scans at early follow-up illustrate the requirement for early detection of treatment efficacy, and clinical implementation relies on a significant impact on treatment management, as repeated examinations increase the radiation burden for the patient. However, the radiation burden is not a major issue for the population presented in this study because of the reduced life expectancy. For this technique to be viable in clinical practice, a technology that can image the entire patient within a short amount of time is needed. A possible answer could be spectral CT (dual-energy CT). A study by Gordic et al. showed a correlation between iodine density measurements for hepatocellular carcinoma and arterial perfusion assessed using DCE-CT [37]. The primary limitation of this study is the small study population, which is illustrated further by the fact that only five patients were scanned with DCE-CT during the entire 12 months period. Furthermore, motion artifacts occurred despite instructions in shallow breathing prior to the scans. The short z-axis of 8 cm of the scanner used when performing DCE-CT often resulted in the assessment of only the primary tumor, and in eight of the thirty-three cases, the entire tumor could not be included in the VOI. As these tumors were visually heterogeneous, they could potentially affect the measurements or calculations of relative changes if not the same part of the tumor was scanned at follow-up. A strength of our study is that it resembles clinical reality and is performed prospectively with a decent amount of follow-up time.

## Conclusion

In conclusion, DCE-CT-identified parameters may serve as potential prognostic biomarkers at baseline and during early treatment in patients with NSCLC treated with immune checkpoint inhibitor therapy. Further research on functional imaging with an increased number of patients with lung cancer is encouraged.

## Data Availability

The datasets generated and/or analyzed during the current study are not publicly available because of further analysis of data for upcoming publications but are available from the corresponding author upon reasonable request.

## Abbreviations

DCE-CT: Dynamic Contrast Enhanced Computed Tomography
BV_decon_: Blood Volume Deconvolution
BF_decon_: Blood Flow Deconvolution
BF_Max slope_: Blood Flow Maximum Slope
OS: Overall Survival
PFS: Progression Free Survival
HR: Hazard Ratio
NSCLC: Non-Small Cell Lung Cancer
iRECIST: Immune Response Evaluation Criteria in Solid Tumors
CE-CT: Contrast Enhanced Computed Tomography
PE: Peak Enhancement
VOI: Volume of Interest
CI95: 95% Confidence Interval

## References

[1] Bray F, Ferlay J, Soerjomataram I, Siegel RL, Torre LA, Jemal A. Global cancer statistics 2018: GLOBOCAN estimates of incidence and mortality worldwide for 36 cancers in 185 countries. CA Cancer J Clin. 2018;68:394–424. 30207593 10.3322/caac.21492

[2] Vokes EE, Ready N, Felip E, Horn L, Burgio MA, Antonia SJ, et al. Nivolumab versus docetaxel in previously treated advanced non-small-cell lung cancer (CheckMate 017 and CheckMate 057): 3-year update and outcomes in patients with liver metastases. Ann Oncol. 2018;29:959–65. 29408986 10.1093/annonc/mdy041

[3] Gettinger S, Horn L, Jackman D, Spigel D, Antonia S, Hellmann M, et al. Five-year follow-up of nivolumab in previously treated advanced non-small-cell lung cancer: results from the CA209-003 study. J Clin Oncol. 2018;36:1675–84. 29570421 10.1200/JCO.2017.77.0412

[4] Seymour L, Bogaerts J, Perrone A, Ford R, Schwartz LH, Mandrekar S, et al. iRECIST: guidelines for response criteria for use in trials testing immunotherapeutics. Lancet Oncol. 2017;18:e143–52. 28271869 10.1016/S1470-2045(17)30074-8 PMC5648544

[5] Monaco L, Gemelli M, Gotuzzo I, Bauckneht M, Crivellaro C, Genova C, et al. Metabolic parameters as biomarkers of response to immunotherapy and prognosis in non-small cell lung cancer (NSCLC): a real world experience. Cancers (Basel). 2021;13:1634. 33915801 10.3390/cancers13071634 PMC8037395

[6] Seban R-D, Assié J-B, Giroux-Leprieur E, Massiani M-A, Soussan M, Bonardel G, et al. Association of the metabolic score using baseline FDG-PET/CT and dNLR with immunotherapy outcomes in advanced NSCLC patients treated with first-line pembrolizumab. Cancers (Basel). 2020;12:2234. 32785166 10.3390/cancers12082234 PMC7463532

[7] Polverari G, Ceci F, Bertaglia V, Reale ML, Rampado O, Gallio E, et al. 18F-FDG pet parameters and radiomics features analysis in advanced nsclc treated with immunotherapy as predictors of therapy response and survival. Cancers (Basel). 2020;12:E1163. 10.3390/cancers12051163 PMC7281558 32380754

[8] Evangelista L, Sepulcri M, Pasello G. PET/CT and the response to immunotherapy in lung cancer. Curr Radiopharm. 2020;13:177–84. 31858908 10.2174/1874471013666191220105449 PMC8206188

[9] Chardin D, Paquet M, Schiappa R, Darcourt J, Bailleux C, Poudenx M, et al. Baseline metabolic tumor volume as a strong predictive and prognostic biomarker in patients with non-small cell lung cancer treated with PD1 inhibitors: a prospective study. J Immunother Cancer. 2020;8:e000645. 32709713 10.1136/jitc-2020-000645 PMC7380842

[10] Park S, Lee Y, Kim T-S, Kim S-K, Han J-Y. Response evaluation after immunotherapy in NSCLC: early response assessment using FDG PET/CT. Medicine (Baltimore). 2020;99:e23815. 33371161 10.1097/MD.0000000000023815 PMC7748304

[11] Niemeijer A-LN, Oprea-Lager DE, Huisman MC, Hoekstra OS, de Wit-van der Veen BJ, et al. Study of 89Zr-pembrolizumab PET/CT in patients with advanced-stage non–small cell lung cancer. J Nucl Med. 2022;63:362–7. Society of Nuclear Medicine. 34272316 10.2967/jnumed.121.261926

[12] Miles KA, Griffiths MR. Perfusion CT: a worthwhile enhancement? Br J Radiol. 2003;76:220–31. 12711641 10.1259/bjr/13564625

[13] Miles KA, Lee T-Y, Goh V, Klotz E, Cuenod C, Bisdas S, et al. Current status and guidelines for the assessment of tumour vascular support with dynamic contrast-enhanced computed tomography. Eur Radiol. 2012;22:1430–41. 22367468 10.1007/s00330-012-2379-4

[14] Ng CS, Kodama Y, Mullani NA, Barron BJ, Wei W, Herbst RS, et al. Tumor blood flow measured by perfusion computed tomography and 15O-labeled water positron emission tomography: a comparison study. J Comput Assist Tomogr. 2009;33:460–5. 19478644 10.1097/RCT.0b013e318182d2e0

[15] Pollard RE, Garcia TC, Stieger SM, Ferrara KW, Sadlowski AR, Wisner ER. Quantitative evaluation of perfusion and permeability of peripheral tumors using contrast-enhanced computed tomography. Invest Radiol. 2004;39:340–9. 15167100 10.1097/01rli.0000124456.82985.35

[16] Mains JR, Donskov F, Pedersen EM, Madsen HHT, Rasmussen F. Dynamic contrast-enhanced computed tomography as a potential biomarker in patients with metastatic renal cell carcinoma: preliminary results from the Danish Renal Cancer Group Study-1. Invest Radiol. 2014;49:601–7. 24691140 10.1097/RLI.0000000000000058

[17] Lind JSW, Meijerink MR, Dingemans A-MC, van Kuijk C, Öllers MC, de Ruysscher D, et al. Dynamic contrast-enhanced CT in patients treated with sorafenib and erlotinib for non-small cell lung cancer: a new method of monitoring treatment? Eur Radiol. 2010;20:2890–8. 20625738 10.1007/s00330-010-1869-5 PMC2978316

[18] Zimmermann S, Peters S, Owinokoko T, Gadgeel SM. Immune checkpoint inhibitors in the management of lung cancer. American Society of Clinical Oncology Educational Book. Philadelphia: Wolters Kluwer; 2018. p. 682–95. 10.1200/EDBK_201319 30231367

[19] Onoi K, Chihara Y, Uchino J, Shimamoto T, Morimoto Y, Iwasaku M, et al. Immune checkpoint inhibitors for lung cancer treatment: a review. J Clin Med. 2020;9:1362. 32384677 10.3390/jcm9051362 PMC7290914

[20] Tunes-da-Silva G, Klein JP. Cutpoint selection for discretizing a continuous covariate for generalized estimating equations. Comput Stat Data Anal. 2011;55:226–35. 22639478 10.1016/j.csda.2010.02.016 PMC3359021

[21] R Core Team. R: a language and environment for statistical computing. Vienna: R Foundation for Statistical Computing; 2020. Available from: https://www.R-project.org/.

[22] Wickham H, Francois R, Kirill M. dplyr: a grammar of data manipulation. 2021. Available from: https://CRAN.R-project.org/package=dplyr.

[23] Therneau TM, Grambsch PM. A package for survival analysis in R. 2000. Available from: https://CRAN.R-project.org/package=survival.

[24] Kassambara A, Kosinski M, Biecek P. survminer: drawing survival curves using “ggplot2”. 2021. Available from: https://CRAN.R-project.org/package=survminer.

[25] Gerds TA. prodlim: product-limit estimation for censored event history analysis. 2019. Available from: https://CRAN.R-project.org/package=prodlim.

[26] Gerds TA, Ozenne B. Publish: format output of various routines in a suitable way for reports. 2020. Available from: https://CRAN.R-project.org/package=Publish.

[27] Dardis C. survMisc: miscellaneous functions for survival data. 2018. Available from: https://CRAN.R-project.org/package=survMisc.

[28] Sudarski S, Shi J, Schmid-Bindert G, Manegold C, Pilz LR, Zhou C, et al. Dynamic volume perfusion computed tomography parameters versus RECIST for the prediction of outcome in lung cancer patients treated with conventional chemotherapy. J Thorac Oncol. 2015;10:164–71. 25247342 10.1097/JTO.0000000000000376

[29] Venkat B, Sharma S, Sharma D, Sood S, Aggarwal N, Sarkar M, et al. CT perfusion in non-small cell lung cancers for assessing treatment response, monitoring treatment and predicting prognosis. Egypt J Radiol Nucl Med. 2018;49:338–45. 10.1016/j.ejrnm.2017.12.007

[30] Wang J, Wu N, Cham MD, Song Y. Tumor response in patients with advanced non-small cell lung cancer: perfusion CT evaluation of chemotherapy and radiation therapy. AJR Am J Roentgenol. 2009;193:1090–6. 19770333 10.2214/AJR.08.1367

[31] Trinidad López C, De La Fuente AJ, OcaPernas R, Delgado Sánchez-Gracián C, Santos Armentia E, VaamondeListe A, et al. Evaluation of response to conventional chemotherapy and radiotherapy by perfusion computed tomography in non-small cell lung cancer (NSCLC). Eur Radiol Exp. 2019;3:23. 31197486 10.1186/s41747-019-0101-x PMC6565789

[32] Drljevic-Nielsen A, Rasmussen F, Mains JR, Thorup K, Donskov F. Baseline blood volume identified by dynamic contrast-enhanced computed tomography as a new independent prognostic factor in metastatic renal cell carcinoma. Transl Oncol. 2020;13:100829. 32653813 10.1016/j.tranon.2020.100829 PMC7350156

[33] Mains JR, Donskov F, Pedersen EM, Madsen HHT, Rasmussen F. Dynamic contrast-enhanced computed tomography-derived blood volume and blood flow correlate with patient outcome in metastatic renal cell carcinoma. Invest Radiol. 2017;52:103–10. 27513367 10.1097/RLI.0000000000000315

[34] Horsman MR, Overgaard J. The impact of hypoxia and its modification of the outcome of radiotherapy. J Radiat Res. 2016;57(Suppl 1):i90–8. 26983987 10.1093/jrr/rrw007 PMC4990104

[35] Festino L, Botti G, Lorigan P, Masucci GV, Hipp JD, Horak CE, et al. Cancer treatment with anti-PD-1/PD-L1 agents: is PD-L1 expression a biomarker for patient selection? Drugs. 2016;76:925–45. 27229745 10.1007/s40265-016-0588-x

[36] Chen Z, Fillmore CM, Hammerman PS, Kim CF, Wong K-K. Non-small-cell lung cancers: a heterogeneous set of diseases. Nat Rev Cancer. 2014;14:535–46. 25056707 10.1038/nrc3775 PMC5712844

[37] Gordic S, Puippe GD, Krauss B, Klotz E, Desbiolles L, Lesurtel M, et al. Correlation between dual-energy and perfusion CT in patients with hepatocellular carcinoma. Radiology. 2016;280:78–87. 26824712 10.1148/radiol.2015151560

